# Device-detected atrial fibrillation in a large remote-monitored cohort: implications for anticoagulation and need for new pathways of service delivery

**DOI:** 10.1007/s10840-023-01481-4

**Published:** 2023-02-03

**Authors:** Catherine J. O’Shea, Anthony G. Brooks, Melissa E. Middeldorp, Curtis Harper, Jeroen M. Hendriks, Andrea M. Russo, James V. Freeman, Rakesh Gopinathannair, Niraj Varma, Thomas F. Deering, Kevin Campbell, Prashanthan Sanders

**Affiliations:** 1 Centre for Heart Rhythm Disorders, University of Adelaide, Adelaide, Australia; 2 Department of Cardiology, Royal Adelaide Hospital, Adelaide, Australia; 3 PaceMate, Bradenton, FL USA; 4 Caring Futures Institute, College of Nursing and Health Sciences, Flinders University, Adelaide, Australia; 5 Cooper Medical School of Rowan University, Camden, NJ USA; 6 Department of Medicine, Yale University School of Medicine, New Haven, CT USA; 7 Kansas City Heart Rhythm Institute, Kansas City, MO USA; 8 Cleveland Clinic, Cleveland, OH USA; 9 Piedmont Heart Institute, Atlanta, GA USA; 10 Health First Heart and Vascular, Melbourne, FL USA

**Keywords:** Atrial fibrillation, Remote monitoring, Anticoagulation, Cardiac implantable electronic device

## Abstract

**Background:**

Remote monitoring (RM) can facilitate early detection of subclinical and symptomatic atrial fibrillation (AF), providing an opportunity to evaluate the need for stroke prevention therapies. We aimed to characterize the burden of RM AF alerts and its impact on anticoagulation of patients with device-detected AF.

**Methods:**

Consecutive patients with a cardiac implantable electronic device, at least one AF episode, undergoing RM were included and assigned an estimated minimum CHA_2_DS_2_-VASc score based on age and device type. RM was provided via automated software system, providing rapid alert processing by device specialists and systematic, recurrent prompts for anticoagulation.

**Results:**

From 7651 individual, 389,188 AF episodes were identified, 3120 (40.8%) permanent pacemakers, 2260 (29.5%) implantable loop recorders (ILRs), 987 (12.9%) implantable cardioverter defibrillators, 968 (12.7%) cardiac resynchronization therapy (CRT) defibrillators, and 316 (4.1%) CRT pacemakers. ILRs transmitted 48.8% of all AF episodes. At twelve-months, 3404 (44.5%) AF < 6 min, 1367 (17.9%) 6 min–6 h, 1206 (15.8%) 6–24 h, and 1674 (21.9%) ≥ 24 h. A minimum CHA_2_DS_2_-VASc score of 2 was assigned to 1704 (63.1%) of the patients with an AF episode of ≥ 6 h, 531 (31.2%) who were not anticoagulated at 12-months, and 1031 (61.6%) patients with an AF episode duration of ≥ 24 h, 290 (28.1%) were not anticoagulated.

**Conclusions:**

Despite being intensively managed via RM software system incorporating cues for anticoagulation, a substantial proportion of patients with increased stroke risk remained unanticoagulated after a device-detected AF episode of significant duration. These data highlight the need for improved clinical response pathways and an integrated care approach to RM.

**Trial registration:**

Australian New Zealand Clinical Trial Registry: ACTRN12620001232921.

**Graphical Abstract:**

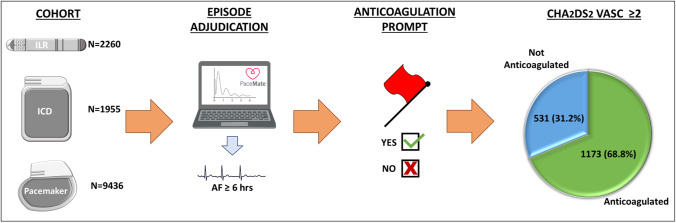

## Introduction

Atrial fibrillation (AF) is associated with a significant risk of ischemic stroke [1]. Device-detected AF has now been correlated with elevated stroke risk [2] independent of the presence or absence of clinical AF symptoms [3]. Anticoagulation reduces both stroke risk and mortality in AF patients. The CHA_2_DS_2_-VASc scoring system correlates risk factors to predict the annual stroke risk in patients with non-valvular AF, and guidelines recommend initiation of anticoagulation in patients with a score of ≥ 2 for men and ≥ 3 for women, and consideration of anticoagulation for patients with a score of 1 for men and 2 for women [4].

Over the past decade, remote monitoring (RM) of cardiac implantable electronic devices (CIEDs) has evolved as a management tool and is now a standard component of device follow-up [5, 6]. RM provides physician access to patient/device information in between clinic visits, in the form of both routine transmissions, which act as a surrogate for an in-person device check, and alerts, which may indicate a patient event, programming concern, or device malfunction. RM alerts facilitate early detection of device-detected arrhythmias, including AF, compared with in-clinic follow-up alone [7, 8]. In the absence of RM, AF would be detected only in the event of a routine follow-up, or an unscheduled encounter pertaining to AF symptoms, heart failure, and embolic event or other untoward clinical events [7]. Early recognition of device-detected AF via RM presents an opportunity to implement anticoagulation and initiate rhythm or rate control strategies in both the asymptomatic patient and the symptomatic patient, who has not yet sought medical attention. Institution of such therapies likely has implications for the associated risks of stroke [4] and heart failure [9] in AF patients.

Using a large clinical cohort of patients undergoing RM via an automated system (PaceMate), we characterize the burden of AF episodes in a remote-monitored CIED population. Specifically, we aimed to determine the impact of AF episodes on rates of anticoagulation, in accordance with CHA_2_DS_2_-VASc scoring.

## Methods

### Data source

For this study, the PaceMate™ RM system was utilized. This system is a partially automated, vendor-neutral RM software system. The system presents RM data acquired from all vendors in a standardized format. The database includes all RM alerts transmitted by CIED patients undergoing RM via PaceMate Live™.

For the study protocol, consecutive AF episodes transmitted via RM from March 2019 until February 2020 were assessed. The study was reviewed and approved by the Human Research Ethics Committees of the Central Adelaide Local Health Network and the University of Adelaide. The study was registered with the Australian New Zealand Clinical Trials Registry (ACTRN12620001232921).

### Study population

Patients were derived from 26 centers in the United States of America and Australia, with devices from multiple manufacturers including Medtronic, Abbott Medical, Boston Scientific, and Biotronik. All patients, who transmitted at least one AF episode with specified episode duration during the twelve-month window, were included and classified according to age, CIED-type, estimated minimum CHA_2_DS_2_-VASc score (details below), and anticoagulation status.

### Protocol for analysis of AF alerts

All AF alerts are received and assessed by device specialists certified by the International Board of Heart Rhythm Examiners (IBHRE). PaceMate device specialists are on-call 24 h a day, 7 days a week for immediate analysis (where possible, depending on the burden of RM transmissions queued for analysis) of RM alerts. After alert analysis, a concise written alert summary is transmitted to patient’s relevant clinic, via an integrated web-based interface, which is streamlined to display alerts from all device manufacturers. The management of incoming alerts/episodes on the PaceMate interface is dictated by each clinic, with allocation of their chosen staff member/s (e.g., cardiac technician, nurse, and physician) to routinely check the interface, and escalation to more senior staff (e.g., physician and electrophysiologist) as per the individualized clinic protocol.

### Classification of device type

CIED-type was classified as either dual-chamber permanent pacemaker (PPM), dual chamber implantable cardioverter-defibrillator (ICD), cardiac resynchronization therapy defibrillator (CRT-D), cardiac resynchronization therapy pacemaker (CRT-P), or implantable loop recorder (ILR). Single-chamber PPMs, leadless PPMs, and single-chamber ICDs were not included in the analysis.

### AF episode duration

AF episodes without an attached episode duration available in the remote transmission were excluded. All remaining AF episodes were classified according to duration. Episodes occurring within a 24-h period were pooled to create a total duration of AF within 24 h. Pooled episodes were then re-classified into one of four pre-specified AF duration windows: (1) less than 6 min during a 24-h period (AF < 6 min), (2) at least 6 min, but less than 6 h during a 24-h period (AF 6 min–6 h), (3) at least 6 h but less than 24 h during a 24-h period (AF 6–24 h), and (4) 24 h or above (AF ≥ 24 h). Patients were then allocated to one of the four AF duration categories, according to their longest pooled episode duration.

### Classification of stroke risk

Patient parameters available to estimate CHA_2_DS_2_-VASc scores included patient age and the presence of a cardiac resynchronization device consistent with heart failure or left ventricular (LV) dysfunction. The RM database did not contain information regarding patient history of hypertension, diabetes mellitus, stroke, vascular disease, or sex.

For the purposes of the study, given the available parameters, we classified the following patients with an AF burden of at least 6 h over 24 h as having an indication for anticoagulation:

1. Patients aged ≥ 75 years; minimum CHA_2_DS_2_-VASc score of 2, due to their age
2. Patients aged ≥ 75 years with a CRT-D or CRT-P in situ; minimum CHA_2_DS_2_-VASc score of 3, due to accrual of 2 points for age, and a third point for the likely presence of underlying LV dysfunction or heart failure, implied by their CIED-type
3. Patients aged 65 to 74 years with a CRT-D, or CRT-P in situ; minimum CHA_2_DS_2_-VASc score of 2, due to accrual of one point for age, and a second point for the likely presence of LV dysfunction or heart failure, implied by their CIED-type
4. Patients aged 65 to 74 years; minimum CHA_2_DS_2_-VASc score of 1, due to their age

### System prompts for anticoagulation and classification of anticoagulation status

Anticoagulation status for each patient was updated following each transmission of an AF episode via bidirectional software system-based communication between PaceMate staff and clinic staff, or by interrogation of the patient’s integrated electronic medical record, which was accessible via the database in some sites. In patients who transmitted an AF episode, whose record did not indicate the current presence of anticoagulation, PaceMate technicians communicated to staff in the relevant clinic the need for consideration of anticoagulation, via the PaceMate web-based interface. Following this communication, clinic staff would respond and the patient’s anticoagulation status would accordingly be updated on the PaceMate user interface. In the absence of a response from clinic staff, PaceMate technicians would continue to re-communicate the anticoagulation query to clinic staff until a response was received, with anticoagulation status clarification. For the purposes of our analysis, anticoagulation status of each patient was as per the PaceMate interface at the close of the 12-month monitoring period.

### Statistical analysis

Continuous data are expressed as the mean ± standard deviation or median (1st and 3rd quartiles). Categorical data are presented as absolute values and percentages. Tests for significance were conducted using the chi-squared test or Fisher’s exact test for categorical variables. A *p* value of ≤ 0.05 was considered significant. All analyses were performed using commercial software (SPSS version 26.0®, SPSS, Inc., Chicago 5 IL, USA).

## Results

In total, 416,360 consecutive AF episodes from 7988 patients were transmitted via remote monitoring during the twelve-month window from March 2019 until February 2020. After exclusion of AF episodes of unspecified duration, 389,188 (93.5%) episodes from 7651 (95.8%) individual patients were included in the analysis.

### AF alert cohort

**Fig. 1:**
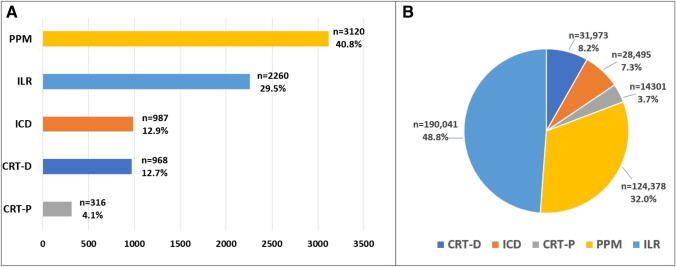
Panel A demonstrates the device types in the cohort. Panel B demonstrates the proportion of AF episodes per device type. PPM, permanent pacemaker; CRT-P, cardiac resynchronization therapy pacemaker; ICD, standard implantable cardioverter defibrillator; CRT-D, cardiac resynchronization therapy defibrillator; ILR, implantable loop recorder

The final AF alert cohort of 7651 patients included 3120 (40.8%) dual-chamber PPMs, 2260 (29.5%) ILRs, 987 (12.9%) dual-chamber ICDs, 968 (12.7%) CRT-Ds, and 316 (4.1%) CRT-Ps (central figure, Fig. 1, Table 1). Of the 389,188 AF episodes transmitted, 190,041 alerts (48.8%) were transmitted by ILRs, 124,378 alerts (32.0%) by PPMs, 31,973 alerts (8.2%) by CRT-Ds, 28,495 alerts (7.3%) by ICDs, and 14,301 alerts (3.7%) by CRT-Ps (Fig. 1).

**Table 1:** Baseline cohort characteristics

Device type	Number (%)	Average age (years)
PPM	3436 (44.9)	77 ± 11
Dual-chamber PPM	3120 (40.8)	77 ± 11
CRT-P	316 (4.1)	78 ± 12
ICD	1955 (25.6)	71 ± 12
Dual-chamber ICD	987 (12.9)	70 ± 12
CRT-D	968 (12.7)	73 ± 12
ILR	2260 (29.5)	70 ± 12
All devices	**7651 (100)**	**74 ± 12**

Values in bold represent total number and overall average age of cohort

*AF*, atrial fibrillation; *PPM*, permanent pacemaker; *CRT-P*, cardiac resynchronization therapy pacemaker; *ICD*, implantable cardioverter defibrillator; *CRT-D*, cardiac resynchronization therapy defibrillator; *S-ICD*, subcutaneous implantable cardioverter defibrillator; *ILR*, implantable loop recorder

### Device manufacturers

The cohort included patients with devices from Medtronic, Abbott Medical, Boston-Scientific, and Biotronik. Medtronic devices accounted for 56.1% (*n* = 4293) of the cohort, Boston Scientific devices for 26.2% (*n* = 2001), Abbott Medical devices for 11.4% (*n* = 875), and Biotronik devices for 4.0% (241) (Table 2).

**Table 2:** Device types according to manufacturer

Device type	Device manufacturer	*n* (%)
PPM	Medtronic	1807
	Boston Scientific	1168
	St. Jude Medical (Abbott)	150
	Biotronik	311
ICD	Medtronic	826
	Boston Scientific	833
	St. Jude Medical (Abbott)	140
	Biotronik	156
ILR	Medtronic	1660
	St. Jude Medical (Abbott)	585
	Biotronik	15
All CIEDs	Medtronic	4293 (56.1)
	Boston Scientific	2001 (26.2)
	St. Jude Medical (Abbott)	875 (11.4)
	Biotronik	482 (6.3)

*PPM*, permanent pacemaker; *ICD*, implantable cardioverter defibrillator; *ILR*, implantable loop recorder; *CIED*, cardiac implantable electronic device

### AF episode duration

After calculation of the longest aggregate AF burden within 24 h per patient, 3404 (44.5%) patients had AF < 6 min, 1367 (17.9%) patients had AF 6 min–6 h, 1206 (15.8%) patients had AF 6–24 h, and 1674 (21.9%) patients had AF ≥ 24 h. This equated to 2880 (37.6%) patients having at least 6 h of AF within a 24-h period.

### Anticoagulation rates in patients aged ≥ 75 years (minimum CHA_2_DS_2_-VASc score of 2) with AF episode at least 6 h in duration

**Fig. 2:**
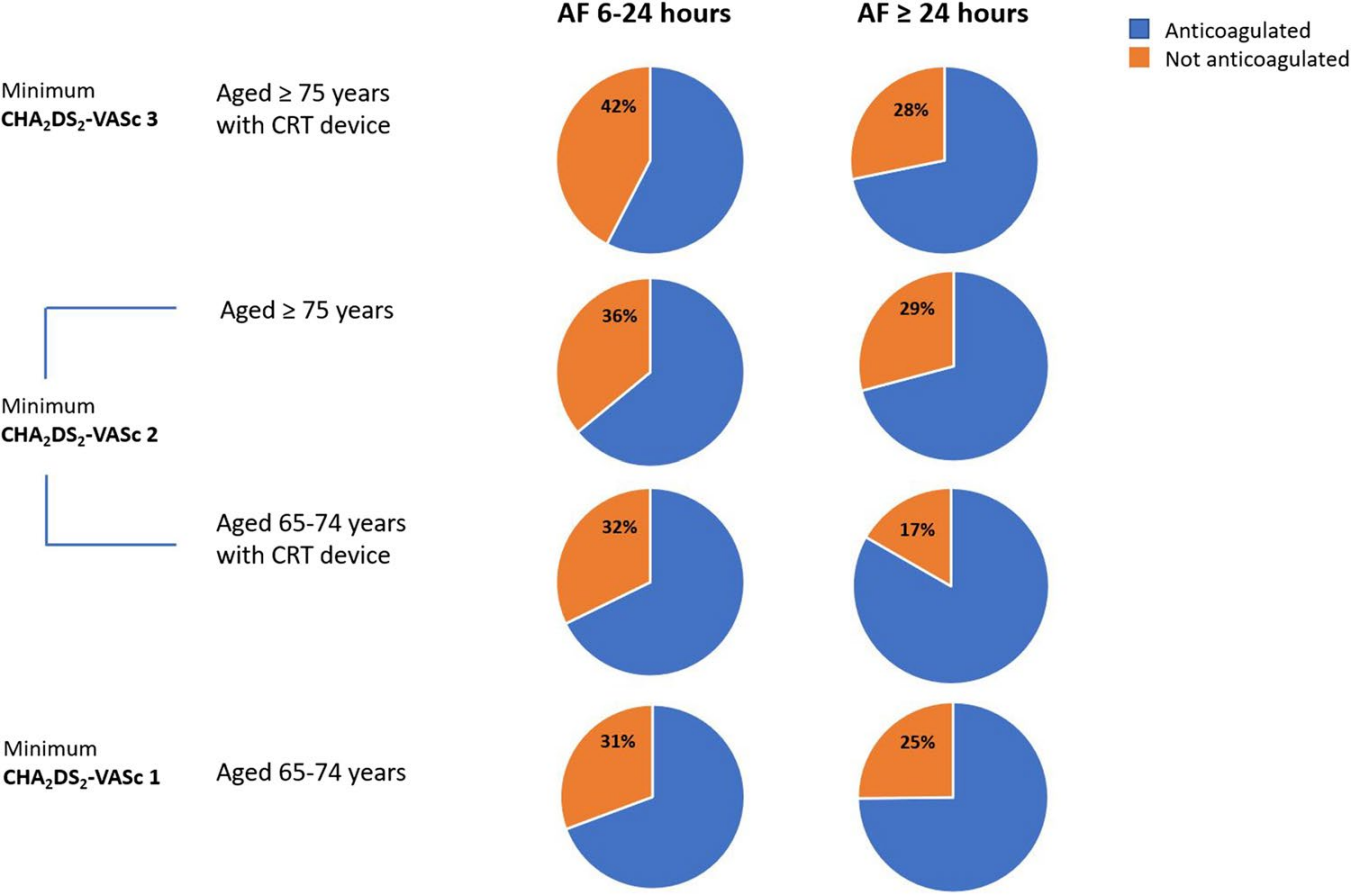
Demonstrates anticoagulation rates in patients with an AF episode of at least 6 h in duration, according to minimum CHA_2_DS_2_-VASc score

Of the 7651 total patients who transmitted AF episodes, 3841 (50.2%) were aged 75 years or above, with a minimum CHA_2_DS_2_-VASc score of 2. Of these patients, 642 (16.7%) had AF durations between 6–24 h and 231 (36.0%) of these patients were not anticoagulated. A further 947 (24.7%) patients aged at least 75 years had AF ≥ 24 h and 276 (29.1%) of these patients were not anticoagulated (Fig. 2).

In patients aged at least 75 years with an AF episode of at least 6 h, 1082 (68.1%) of a total 1589 patients were receiving anticoagulation at the end of the 12-month monitoring period, while 507 (31.9%) were not.

Of the 3841 patients aged ≥ 75 years, 682 had a CRT-D or CRT-P in situ, elevating their minimum CHA_2_DS_2_-VASc score to 3. Of these patients, 92 had AF 6–24 h with 39 (42.4%) not anticoagulated. A further 234 patients had AF ≥ 24 h with 66 (28.2%) not anticoagulated (Fig. 2).

Of the total 326 patients designated a minimum CHA_2_DS_2_-VASc score of 3, due to a combination of their age and device type, who transmitted an AF episode of at least 6 h in duration, there were 221 (67.8%), who were receiving anticoagulation and 105 (32.2%) who were not.

### Anticoagulation rates in patients aged 65–74 years with AF episodes at least 6 h in duration

A total of 2051 patients in the cohort were aged 65–74 years, with a minimum CHA_2_DS_2_-VASc score of 1. Of these patients, 339 had AF 6–24 h and 104 (30.7%) were not anticoagulated. A further 449 patients had AF ≥ 24 h and 113 (25.2%) were not anticoagulated (Fig. 2). When all patients aged 65–74 years with an AF episode of at least 6 h were pooled, 517 (72.5%) of a total 788 patients were receiving anticoagulation, while 217 (27.5%) were not.

Of the 2051 patients aged 65–74 years, 313 had a CRT-D or CRT-P in situ, elevating their minimum CHA_2_DS_2_-VASc score to 2. Of these patients, 31 had AF 6–24 h, 10 (32.3%) of whom were not anticoagulated. A further 84 patients had AF ≥ 24 h and 14 (16.7%) were not anticoagulated (Fig. 2).

Of the total, 115 patients aged 65–74 years with a CRT-D or CRT-P in situ, and an AF episode of at least 6 h, 91 (79.1%) were receiving anticoagulation, while 24 (20.9%) were not.

### Comparison of anticoagulation rates across patient age groups

**Fig. 3:**
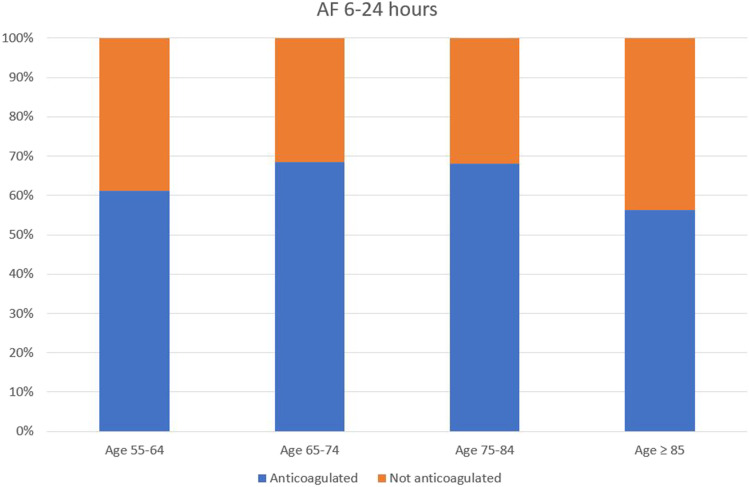
Demonstrates anticoagulation status across different age brackets, in patients with maximum AF episode duration of 6 to 24 h

**Fig. 4:**
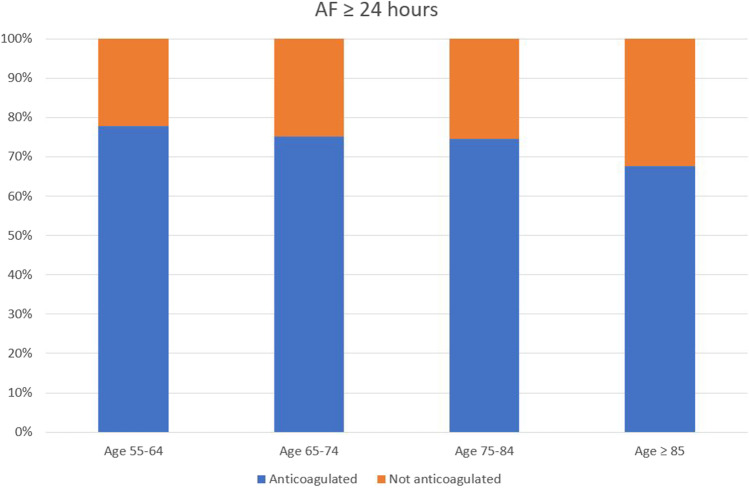
Demonstrates anticoagulation status across different age brackets, in patients with maximum AF episode duration of more than 24 h

Use of anticoagulation was compared across age categories in all patients aged at least 55 and above, with an AF episode duration of at least 6 h. In patients with AF 6–24 h, anticoagulation was present in 61.2% of those aged 55–64 years, 68.5% of those aged 65–74 years, 68.0% of those aged 75–84 years, and 56.4% of those aged ≥ 85 years (Fig. 3). In patients with AF ≥ 24 h, anticoagulation was present in 77.8% of those aged 55–64 years, 75.2% of those aged 65–74 years, 74.5% of those aged 75–84 years, and 67.6% of those aged ≥ 85 years (Fig. 4).

## Discussion

### Major findings

Early detection of AF and the potential opportunity to intervene to reduce stroke risk is a key benefit of RM. Using a large clinical RM database across multiple centers, we identified the following with regards to AF episodes:

• AF episode burden was significant across all device types; however, there was an over-representation of AF episodes detected by ILRs
• A substantial proportion of patients with a significant risk of ischemic stroke as indicated by their AF episode duration combined with their CHA_2_DS_2_-VASc score remain not anticoagulated
• Anticoagulation rates among older patients were comparatively lower than rates among younger patients

### Burden of AF alerts

A large proportion (48.8%) of AF episodes were transmitted by ILRs, despite ILRs accounting for only 29.5% of the cohort. This may be partially attributable to the nature of ILR indications, including syncope [10, 11], cryptogenic stroke [12–14], and palpitations [15, 16], all of which may be caused by AF or its termination. Alternatively, this may represent transmission of false-positive episodes, which are a common occurrence in ILRs, largely due to atrial ectopy or noise [17].

By comparison, despite accounting for the bulk of CIEDs (44.9%) in the cohort, PPMs were responsible for only 35.7% of AF episodes, while defibrillator patients represented 25.6% of the cohort but transmitted only 15.5% of AF episodes. Device programming in PPMs and ICDs may further explain this between-device group disparity; patients with known AF, on appropriate therapy, may be programmed with a longer AF duration threshold for triggering of an RM alert.

### AF episode duration and anticoagulation

While device-detected AF has been established as a risk factor for stroke, questions remain regarding the appropriate AF episode duration threshold for commencement of anticoagulation. A sub-analysis of the ASSERT study demonstrated a significantly increased risk of stroke or systemic embolism in patients with a device-detected AF episode of at least 24 h, with shorter episodes (6 min–24 h) not correlating with an increase in risk [18]. Another study of over 9000 CIED patients over a ten-year period showed device-detected AF of at least 5.5 h was associated with heightened stroke risk [19]. With regards to shorter duration episodes, the LOOP study anticoagulated high-risk patients with at least 6 min of AF detected on a loop recorder and found no significant reduction in stroke of systemic embolism compared to a control group [20]. A recent meta-analysis of studies reporting stroke events in patients with device-detected AF demonstrated a low risk of stroke (0.93 per 100 person years) in association with “short” duration AF episodes [21]; however, there was significant between-study heterogeneity in the definition of such episodes, ranging from below 6 min duration [3] to below 5.5 h burden in one day [22]. The same meta-analysis showed that overall, device-detected AF was associated with an increased stroke risk (absolute annual risk 1.89 per 100 person years, increased to 2.76 per 100 person years in case of mean CHADS_2_ score 2.1) [21].

Al-Gibbawi et al. assessed stroke/transient ischemic stroke (TIA) incidence in a high-risk unanticoagulated CIED patient population with an average CHA_2_DS_2_-VASc score of 4.1 and a 19.9% rate of previous TIA/stroke. They found no correlation between longest AF episode duration and risk of stroke, nor between overall AF burden and risk of stroke[23]. Comparatively, Go et al. demonstrated an association between AF burden determined by wearable cardiac monitors and risk of stroke, in a population with a lower average CHA_2_DS_2_-VASc score (2.6), and a lower rate of previous TIA/stroke (2.7%) [24]. These contrasting findings raise the possibility that in higher-risk patients (as determined by CHA_2_DS_2_-VASc scores) with a CIED in situ, AF burden and episode duration are less predictive of stroke [23]. In our cohort, such high-risk patients with CHA_2_DS_2_-VASc scores of at least 3, with AF of at least 6 h, but less than 24 h, were anticoagulated at a rate of only 57.6%.

### Rates of anticoagulation in patients with CHA_2_DS_2_-VASc score ≥ 2

The European Heart Rhythm Association guidelines recommend oral anticoagulation in patients with a CHA_2_DS_2_-VASc score ≥ 2 in the presence of an AF burden of more than 5.5 h in one day [25]. Our analysis demonstrates that among patients with a transmitted AF episode of at least 6 h, and a minimum CHA_2_DS_2_-VASc score of 2, the overall anticoagulation rate was 68.8% (central figure). Kaplan et al. looked specifically at device-detected AF in a large real-world remote-monitored CIED patient population and described comparatively low rates of anticoagulation. In patients with daily AF burden of between 6 min and 23.5 h, anticoagulation was prescribed in 14.1%, 29.6%, and 44.7% of those with CHA_2_DS_2_-VASc scores of 2, 3–4, and ≥ 5, respectively. These rates may be explained by inclusion of short-duration episodes for which treating clinicians opted against anticoagulation; however, anticoagulant use remained low in patients with longer duration AF (> 23.5 h), being prescribed in 24.3%, 45.3%, and 67.2% of those with CHA_2_DS_2_-VASc scores of 2, 3–4, and ≥ 5, respectively. [26]. In contrast, Steinberg et al. compared rates of oral anticoagulation in over 60,000 patients with newly diagnosed clinical AF within two AF registries and found anticoagulation use in 69% (GARFIELD-AF (international)) and 87% (ORBIT-AF II (US-only)) of patients with a CHA_2_DS_2_VASc score ≥ 2 [27]. The discrepancy in anticoagulation rates may be reflective of physician reluctance in prescribing anticoagulation for patients with device-detected AF, especially as US guidelines do not specify thresholds for the use of anticoagulation in this patient cohort [28]. Given the uncertain benefits of anticoagulation among patients with device-detected AF, particularly in those with episodes shorter than 24 h in duration, outcomes from pending trials (e.g., ARTESIA (NCT01938248) [29] and NOAH-AFNET 6 (NCT02618577)) [30] will hopefully provide further guidance regarding this issue.

Although the CHA_2_DS_2_-VASc scoring system was not derived for risk stratification specifically in device-detected AF [31], subclinical AF is a known strong predictor of clinical AF [21], and stroke risk in modern AF cohorts is similar to risk in device-detected AF [21]. Thus, it is not unreasonable to estimate a 2.9% annual risk of stroke, TIA, or systemic embolism in accordance with a CHA_2_DS_2_-VASc score of 2 [32]; our analysis left 31.2% of patients in this category without anticoagulation.

### Rates of anticoagulation among younger versus older patients

The lowest anticoagulation rates for AF episodes of significant duration were seen in older patients, aged 75 or above. Of these patients with an AF episode of between 6 and 24 h in duration, 64.0% were anticoagulated. In patients aged ≥ 75 years with a CRT device in situ, the anticoagulation rate for an AF episode between 6 and 24 h was even lower, at 57.6%, despite a minimum CHA_2_DS_2_-VASc score of 3, inferring an approximate 3.2% annual risk of ischemic stroke, and 4.6% annual risk of stroke/TIA/systemic embolism [32]. Anticoagulation rates among patients aged 65–74 years were comparatively higher at 72.5% in those with an AF episode of at least 6 h in duration and as high as 74.8% in those with AF ≥ 24 h. When comparing younger patients in the cohort (age 55–64 years) and older patients (aged at least 85 years) with AF of at least 6 h in duration, we unexpectedly demonstrated higher anticoagulation rates in the younger patients, compared with their older counterparts (61.2% vs. 56.4% for AF 6–24 h, and 77.8% vs. 67.6% for AF 24 h).

Lower anticoagulation rates in older patients, with higher CHA_2_DS_2_-VASc scores, may be attributable to physician or patient concerns around frailty, falls risk, and bleeding risk, as this demographic tends to also have higher HAS-BLED scores. Studies have shown, however, that the net clinical benefit of anticoagulation in AF patients exceeds the risk of significant bleeding in most patients [33, 34]. In a large cohort study of over 180,000 AF patients, Friberg et al. identified only 0.4% of patients with a CHA_2_DS_2_-VASc score of ≥ 1 in whom bleeding risk exceeded ischemic stroke risk, with most patients not exhibiting a true contraindication to anticoagulation [33]. Another factor contributing to lower use of anticoagulation in the elderly demographic may be the use of left atrial appendage occlusion to negate the requirement for anticoagulation.

### Anticoagulation rates in patients with long duration AF episodes (≥ 24 h)

Device-detected AF episode duration of ≥ 24 h has been established as an independent risk factor for embolic events [18, 35]; Botto et al. found episodes of ≥ 24 h in duration to infer an annual embolic event risk of up to 4%, even in patients with a CHADS_2_ score of only 1 [36]. The study utilized CHADS_2_ scores only; however, some patients may have had a higher CHA_2_DS_2_-VASc score if calculated. More recently, Kaplan et al. assessed the occurrence of stroke and systemic embolism in over 20,000 unanticoagulated CIED patients and established both increasing AF episode duration and increasing CHA_2_DS_2_-VASc score to be associated with a heightened embolic risk. Importantly, in patients with long AF episode duration (≥ 23.5 h) and a CHA_2_DS_2_-VASc score of 1, the annual incidence of stroke/systemic embolism was only 0.56% [37], suggesting that long-duration device-detected AF episodes may not warrant anticoagulation in CHA_2_DS_2_-VASc-1 patients. Despite this, our study showed that among patients with AF ≥ 24 h, those aged less than 65 years, and those aged 65–74 years, had relatively high anticoagulation rates of 74.9% and 74.8%, respectively. In some patients, this may be reflective of the presence of factors unknown to us, elevating their CHA_2_DS_2_-VASc score. Again, use of anticoagulation was comparatively lower (70.9%) in patients aged ≥ 75 years, perhaps reflecting physician confidence regarding low bleeding risk in the younger cohort.

## Clinical implications

The findings of this study highlight the need for strategies to address the ischemic stroke/systemic embolism risk that remains unaddressed in a significant proportion of patients with device-detected AF. Education regarding stroke risk in patients with subclinical AF and the net clinical benefit of anticoagulation in high-risk patients provides one with potential opportunity to manage better this patient cohort. Furthermore, implementation of structured RM clinical response pathways and integrated care, to promote consideration of anticoagulation, is required. Given the burden that AF episodes place on current RM pathways, systems can be re-designed with inclusion of automated features to prompt anticoagulation decisions as an alternative to reliance on human prompts.

## Limitations

Our data were derived from a large remote monitoring database with a limited number of baseline characteristics. CHA_2_DS_2_-VASc scores could not be definitively obtained due to unknowns regarding the presence of hypertension, diabetes mellitus, heart failure/left ventricular dysfunction, and vascular disease in the cohort. Further, we did not have access to sex in a significant proportion of the cohort. Our analysis was performed based on minimum CHA_2_DS_2_-VASc scores according to the available patient characteristics. This likely lead to an underestimate of patients in whom stroke risk crossed the threshold to justify anticoagulation. In the absence of data regarding procedural interventions, we cannot exclude to possibility that some unanticoagulated patients in our study population have undergone left atrial appendage occlusion and are thus appropriately without anticoagulation. Though anticoagulation status was assessed only in those patients with a burden of at least 6 h within a 24 h period, meaning the likelihood of a false-positive AF episode is minimal [38], in the absence of individual electrogram review, we cannot definitively exclude the possibility of inclusion of some non-AF rhythms.

## Conclusion

Despite participation in an intensively managed RM system with in-built cues for consideration of anticoagulation, more than 30% of patients with device-detected AF were not anticoagulated in the presence of a risk factor profile inferring a significant risk of ischemic stroke or systemic embolism. Older patients, aged 75 or above, were under-anticoagulated compared with their younger counterparts. These data represent potential missed opportunities to implement pharmacotherapy for stroke prevention. Improved RM clinical response pathways are required to ensure adherence to stroke prevention measures in the device-detected AF population.

## Abbreviations

AF: Atrial fibrillation
CIED: Cardiac implantable electronic device
CRT: Cardiac resynchronization therapy
IBHRE: International Board of Heart Rhythm Examiners
ICD: Implantable cardioverter defibrillator
ILR: Implantable loop recorder
LV: Left ventricle
PPM: Permanent pacemaker
RM: Remote monitoring
TIA: Transient ischemic stroke
